# Using Electronic Health Records to Classify Cancer Site and Metastasis

**DOI:** 10.1055/a-2544-3117

**Published:** 2025-06-18

**Authors:** Kurt Kroenke, Kathryn J. Ruddy, Deirdre R. Pachman, Veronica Grzegorczyk, Jeph Herrin, Parvez A. Rahman, Kyle A. Tobin, Joan M. Griffin, Linda L. Chlan, Jessica D. Austin, Jennifer L. Ridgeway, Sandra A. Mitchell, Keith A. Marsolo, Andrea L. Cheville

**Affiliations:** 1 Department of Medicine, Indiana University School of Medicine, Indianapolis, Indiana, United States; 2 Regenstrief Institute, Inc., Indianapolis, Indiana, United States; 3 Division of Medical Oncology, Mayo Clinic, Rochester, Minnesota, United States; 4 Division of Community Internal Medicine, Geriatrics, and Palliative Care, Mayo Clinic, Rochester, Minnesota, United States; 5 Department of Physical Medicine and Rehabilitation, Mayo Clinic, Rochester, Minnesota, United States; 6 Department of Internal Medicine, Yale University School of Medicine, New Haven, Connecticut, United States; 7 Robert D. and Patricia E. Kern Center for the Science of Health Care Delivery, Mayo Clinic, Rochester, Minnesota, United States; 8 Division of Health Care Delivery Research, Mayo Clinic, Rochester, Minnesota, United States; 9 Division of Nursing Research, Department of Nursing, Mayo Clinic College of Medicine and Science, Mayo Clinic, Rochester, Minnesota, United States; 10 Department of Epidemiology, Mayo Clinic College of Medicine and Science, Scottsdale, Arizona, United States; 11 Outcomes Research Branch, Healthcare Delivery Research Program, Division of Cancer Control and Population Sciences, National Cancer Institute, Rockville, Maryland, United States; 12 Department of Population Health Sciences, Duke University School of Medicine, Durham, North Carolina, United States

**Keywords:** neoplasms, cancer site, metastasis, pragmatic clinical trial, electronic health records, natural language processing, cancer registry

## Abstract

**Background:**

The Enhanced EHR-facilitated Cancer Symptom Control (E2C2) Trial is a pragmatic trial testing a collaborative care approach for managing common cancer symptoms. There were challenges in identifying cancer site and metastatic status.

**Objectives:**

This study compares three different approaches to determine cancer site and six strategies for identifying the presence of metastasis using EHR and cancer registry data.

**Methods:**

The E2C2 cohort included 50,559 patients seen in the medical oncology clinics of a large health system. SPPADE symptoms were assessed with 0 to 10 numeric rating scales (NRS). A multistep process was used to develop three approaches for representing cancer site: the single most prevalent International Statistical Classification of Diseases and Related Health Problems, 10th Revision (ICD-10) code, the two most prevalent codes, and any diagnostic code. Six approaches for identifying metastatic disease were compared: ICD-10 codes, natural language processing (NLP), cancer registry, medications typically prescribed for incurable disease, treatment plan, and evaluation for phase 1 trials.

**Results:**

The approach counting the two most prevalent ICD-10 cancer site diagnoses per patient detected a median of 92% of the cases identified by counting all cancer site diagnoses, whereas the approach counting only the single most prevalent cancer site diagnosis identified a median of 65%. However, agreement among the three approaches was very good (kappa > 0.80) for most cancer sites. ICD and NLP methods could be applied to the entire cohort and had the highest agreement (kappa = 0.53) for identifying metastasis. Cancer registry data was available for less than half of the patients.

**Conclusion:**

Identification of cancer site and metastatic disease using EHR data was feasible in this large and diverse cohort of patients with common cancer symptoms. The methods were pragmatic and may be acceptable for covariates, but likely require refinement for key dependent and independent variables.

## Background and Significance

Symptoms and functional impairment are a prominent source of distress and diminished quality of life in persons with cancer. 1 2 3 4 Additionally, cancer-related symptoms contribute to decreased work productivity along with increased health care utilization and costs. 5 6 7 Sleep disturbance, pain, physical function impairment, anxiety, depression, and energy deficit/fatigue (SPPADE) are a particularly common set of symptoms that have high rates of co-occurrence, undertreatment, and persistence in cancer survivors. 5 8 9 10

The Enhanced EHR-facilitated Cancer Symptom Control (E2C2) trial is a stepped wedge, cohort cluster randomized, pragmatic clinical trial designed to evaluate the effectiveness of routine symptom surveillance and guideline-informed symptom management targeting six SPPADE symptoms (clinicaltrials.gov identifier: NCT03892967). 11 A large, population-level cohort of patients was assembled from all medical oncology clinics of a large regional healthcare system. Baseline patient characteristics and outcomes were extracted from a common Epic electronic health record (EHR). Challenges of using the EHR for clinical research in cancer and other conditions have been described. 12 Extracting and operationalizing key clinical variables for E2C2 required the development and comparison of multistep algorithmic approaches.

Assembly of the E2C2 trial cohort revealed important challenges in using EHR data to classify two essential cancer characteristics: cancer site and metastatic status. First, individuals not uncommonly may have more than one site of cancer in which case methods used to study single sites of cancer may require modification when applied to patients with cancer at multiple sites. Second, a common assumption is that cancer registries are the most reliable and comprehensive source of data, particularly at the time of diagnosis. However, many patients with cancer seen at a tertiary center are not necessarily included in that center's registry and, importantly, registry data reflects the stage of cancer at initial diagnosis and is typically not updated to capture the longitudinal trajectory of the disease including progression to more advanced stages. 13 14 15 16 Third, the increasing emphasis on using real-world data for pragmatic trials and other types of clinically embedded research requires informatics-based strategies for classifying important constructs such as cancer site and metastatic status. 17 18

## Objectives

Data were derived from a cohort of more than 50,000 patients with cancer participating in the E2C2 pragmatic trial of symptom management. The objectives in this study are: (1) to compare three approaches for using EHR data to classify cancer site; (2) to compare six strategies for determining the likelihood of cancer metastasis. The ultimate aim is to balance data precision with pragmaticism, consider data's fitness for use, and offer means of optimizing EHR variable specification in large pragmatic trials as well as other types of embedded research including learning health care systems. 17 18

## Methods

Cohort clusters from four adjacent health system regions and oncology care teams at a large academic medical center, all part of a large, multi-state health system, were randomly assigned to implement the E2C2 intervention at sequential 8-month intervals. Participants in this stepped wedge trial were identified using an EHR algorithm that required patients to have been assigned a diagnostic code included in the Epic foundation system's “All Cancers Grouper,” a clinical encounter with a medical oncology clinician, and an encounter visit type corresponding to an initial or follow-up medical oncology evaluation. Patients with cancer receiving care at the study sites were administered patient-reported outcome measures (PROM) to report SPPADE symptoms electronically, results of which were used to determine the level of intervention among clusters that had gone live with the intervention. The intervention included automated self-management support for patients reporting moderate (≥ 4 on 0-10 scale) levels of ≥1 SPPADE symptom and collaborative care model (CCM)-based management for patients reporting severe (≥7) symptoms. E2C2 was approved as a minimal risk study by the Mayo Clinic Institutional Review Board; the requirement for informed consent was waived. Additional details of the E2C2 trial protocol are reported elsewhere. 11 19

## Determining Cancer Site

**Fig. 1:**
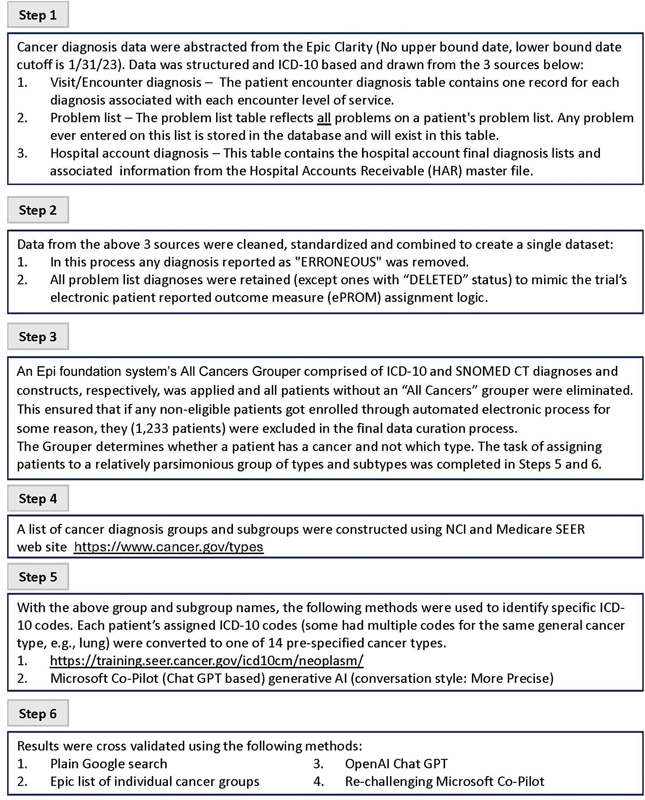
Algorithm for using electronic health record (EHR) data to determine cancer site.

Three methods were used to classify cancer site into 14 specific categories using the combined dataset of patient diagnoses from medical oncology and other clinical encounters, hospital visits, and the EHR problem list. Excluded from all approaches were diagnoses in the categories “Other solid tumor with distant metastatic,” “Neoplasm of uncertain or unspecific behavior,” “Metastatic,” “Other solid tumor,” “Benign neoplasm,” “Unspecified,” and “Lymph node disease.” The algorithm for determining cancer site is detailed in Fig. 1 . The All Cancers Grouper in Step 3 is a list of every diagnosis inside Mayo's instance of Epic that a provider might use. These are used in office visits, admissions, and problem lists to record when a patient has a particular condition and is a vital and routine part of care. This is built using the SNOMED CT hierarchy—specifically the “Malignant neoplastic disease” concept and all child concepts. Any diagnoses in Mayo's instance of Epic associated with this concept or one of its branches is included in this grouper.

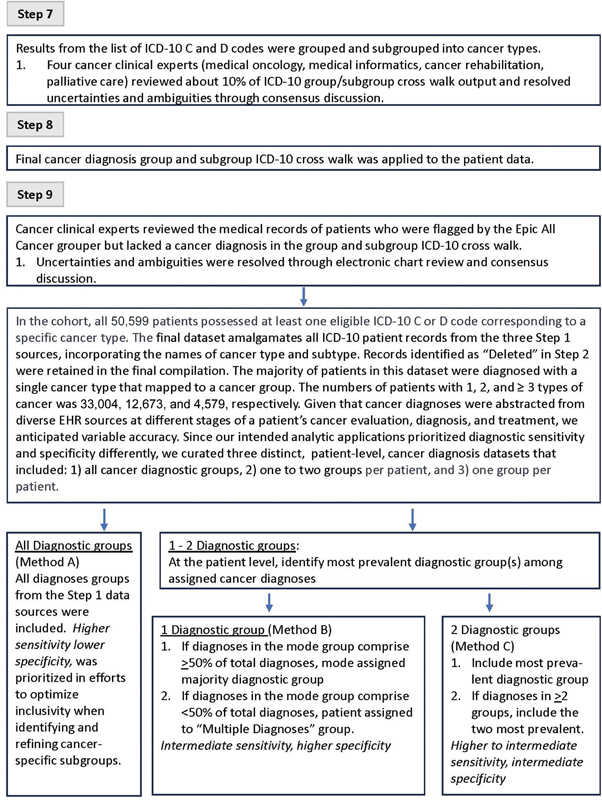



*Method A* (most sensitive) counted all categories coded for a patient with no limit on the number of cancer sites. *Method B* (most specific) allowed only one primary diagnosis category per patient. For patients with two or more diagnosis categories, the one that made up >50% of diagnoses was assigned. If there was no majority diagnosis, the category assigned was “multiple.” If there were no specific valid diagnosis categories, the category assigned was “nonspecific.” *Method C* (intermediate sensitivity/specific) allowed up to two primary diagnosis categories (the two most common out of all that were coded). For those with three or more cancer sites, the two most commonly coded cancer site diagnoses were assigned.

## Determining Metastatic Status

Six approaches to classifying cancer as metastatic are detailed in Table 1 . Two methods of classification that could be applied to all patients were considered primary approaches. Four other methods were considered secondary approaches.

**Table 1:** Details of methods used to determine metastatic status in E2C2 trial cohort

Method	*N* a	Metastatic	Comments
ICD diagnoses	50,559	22,461 44.4%	• Includes a group of diagnoses which are indicative of metastatic illness. • All patients in the cohort possess diagnosis data because a positive cancer diagnosis is required for inclusion in the cohort. • Diagnoses indicating regional lymph node involvement were not included. • Diagnoses were filtered to the time period of 1 year before inception of the study to the end of the study. • Diagnoses on the problem list that were listed as “Deleted” were removed, as this indicates that they were entered in error. • Patients with metastatic diagnoses had between 1 and 1,004 diagnoses indicating metastatic disease (mean = 28.43, median = 8).
Natural language processing (NLP)	50,559	22,336 44.2%	• All patients were run through a natural language processing (NLP) program. • Details of the NLP program are provided in the footnote. **b**
Cancer registry	24,160	5,914 24.5%	• Patients typically have metastatic disease assessed during intake to the registry at the time of diagnosis, but the registry does not consistently include follow-up information about progression to metastatic disease after diagnosis. • Therefore, the registry is reasonably accurate regarding metastatic disease at the time of diagnosis, but does not reliably identify patients who subsequently go on to develop metastatic disease after diagnosis.
Treatment plan	15,678 27,460	10,328 65.9% 10,328 37.6%	• 27,460 of the patients have a Beacon module treatment plan. • Of the patients with treatment plans, 15,678 had a treatment goal listed. • Of the patients who had a treatment goal listed, 10,328 had at least one treatment plan with the goal stated as “Palliative” or “Control.” Specification of either of these treatment goals was considered to indicate metastatic disease. • However, having other treatment goals specified in the Beacon treatment plan is not an indicator that metastatic disease is not present.
Medications	48,647	5,300 10.9%	• 48,647 of the sample had medication prescription orders and medication administrations data (including outpatient and inpatient data). • Of these, we have information on certain medications used to treat incurable types of cancers (brain, breast, gastrointestinal, genitourinary, hematologic, lung, leukemia, lymphoma, sarcoma). • Other types of cancer do not have this data. Some of the commentary on medications indicated that the cancer was always considered incurable, or pointed to procedures that are performed for that specific type of cancer. • 42,863 had at least one diagnosis of a valid type of cancer for this analysis. • Of these, 5,300 were taking medication that indicated metastatic disease.
Clinical trial	1,050	1,050 100%	• Encounter descriptions with the phrase “Trial” were identified. • Of these, 1,050 had phase I trial clinic encounter.

a *N* is the number of individuals for which data was available for each method.

b A *natural language processing (NLP)* algorithm was applied to EHR data for all E2C2 patients. The NLP algorithm included structured data elements but was additionally applied to unstructured data including all clinical notes, pathology reports, and inpatient documentation from allied health and other staff, among others. The algorithm's construction involved developing rule-based algorithms by integrating expert knowledge engineering and sublanguage analysis based on the Open Health Natural Language Processing (OHNLP) Toolkit implemented across the Mayo Clinic Enterprise. EHR clinical notes and pathology reports were used for metastasis disease information extraction. Specifically, 48 cancer types and corresponding staging knowledge from the American Joint Committee on Cancer (AJCC) Cancer Staging Manual were collected. NLP rules were developed to run on the OHNLP Toolkit. The algorithms on top of the OHNLP Toolkit checked the assertion status of each identified text mention that included certainty (i.e., positive, negative, and possible), temporality (historical or current), and experience (i.e., associated with the patient or someone else). Post-processing was done by exploring the context and structure of clinical texts grammatically and semantically to learn the specialized rules, i.e., “sublanguage.” Finally, the patient-level summary algorithm was developed to derive the presence or absence of patient-level metastatic disease. A review of a subset comprising 500 randomly selected patients from the E2C2 cohort, excluding those with hematological malignancies, revealed a 0.85 recall rate.

### Primary Approaches

1. *ICD-10 codes* included a group of diagnoses that are indicative of metastatic disease. Diagnoses indicating regional lymph node involvement were not included in this designation. Diagnoses were filtered to the time period of 1 year before enrollment to the end of the study (February 2023).
2. A *natural language processing (NLP)* algorithm was applied to EHR data for all E2C2 patients, drawing upon prior research. 20 21 22 23 The NLP algorithm included structured data elements but was additionally applied to unstructured data including all clinical notes, pathology reports, and inpatient documentation from allied health and other staff, among others. Details of the NLP algorithm are provided in the Table 2 footnote.

**Table 2:** E2C2 study cohort

Characteristic	Total sample a *N* = 50,559	Completed ≥1 PROM *N* = 40,005		Did not complete a PROM *N* = 10,554		SMD b
	*N*	*N*	(%)	*N*	(%)	
Age						0.12
<40	3,183	2,570	(6.4)	613	(5.8)	
40–64	21,386	17,367	(43.4)	4,019	(38.1)	
≥65	25,990	20,068	(50.2)	5,922	(56.1)	
Sex						0.09
Female	28,507	22,926	(57.3)	5,581	(52.9)	
Male	22,051	17,078	(42.7)	4,973	(47.1)	
Ethnicity						0.06
Not Hispanic or Latino	49,411	39,318	(98.3)	10,093	(95.6)	
Hispanic or Latino	644	456	(1.1)	188	(1.8)	
Unknown/Not reported	504	231	(0.6)	273	(2.6	
Race						0.15
White	46,952	37,724	(94.3)	9,228	(87.4)	
African-American	836	512	(1.3)	324	(3.1)	
American Indian/Alaska Native	262	195	(0.5)	67	(0.6)	
Asian/Pacific Islander	1,054	778	(1.9)	276	(2.6)	
Other/Unknown	1,455	796	(2.0)	659	(6.2)	
Marital status						0.19
Married/Partnered	34,850	28,386	(71.0)	6,464	(61.3)	
Divorced/Separated	4,553	3,477	(8.7)	1,076	(10.2)	
Widowed	4,746	3,541	(8.9)	1,205	(11.4)	
Single	6,126	4,519	(11.3)	1,607	(15.2)	
Unknown	284	82	(0.2)	202	(1.9)	
Employment						0.19
Employed	19,199	15,918	(39.8)	3,281	(31.1)	
Retired	24,530	19,188	(48.0)	5,342	(50.6)	
Not employed/student/military	4,411	3,216	(8.0)	1,195	(11.3)	
Disabled	2,054	1,556	(3.9)	498	(4.7)	
Unknown	365	127	(0.3)	238	(2.3)	
Payor						0.11
Government	30,773	24,031	(60.1)	6,742	(63.9)	
Non-government	19,281	15,751	(39.4)	3,530	(33.5)	
Unknown	505	223	(0.6)	282	(2.7)	
Education						*0.24
Less than high school	9,173	7,593	(19.0)	1,580	(15.0)	
High school	1,333	1,114	(2.8)	219	(2.1)	
Some college or Associate degree	10,452	9,182	(23.0)	1,270	(12.0)	
Bachelor's degree	7,502	6,707	(16.8)	795	(7.5)	
Master's or Doctoral degree	5,513	4,934	(12.3)	579	(5.5)	
Unknown	16,586	10,475	(26.2)	6,111	(57.9)	
RUCA						0.02
Urban (1–3)	27,931	22,338	(55.8)	5,593	(53.0)	
Micropolitan (4–6)	8,647	6,867	(17.2)	1,780	(16.9)	
Small town (7–9)	6,929	5,477	(13.7)	1,452	(13.8)	
Rural (10)	6,366	5,061	(12.7)	1,305	(12.4)	
Unknown	686	262	(0.7)	424	(4.0)	
Metastatic status c						*0.28
Not metastatic	22,333	16,704	(41.8)	5,629	(53.3)	
Metastatic	16,554	14,084	(35.2)	2,470	(23.4)	
Uncertain	11,672	9,217	(23.0)	2,455	(23.3)	
Cancer type d						
Breast	10,611	8,760	(21.9)	1,851	(17.5)	0.11
Endocrine	1,386	1,123	(2.8)	263	(2.5)	0.02
Gastrointestinal	12,774	9,522	(23.8)	3,252	(30.8)	0.16
Genitourinary	4,556	3,645	(9.1)	911	(8.6)	0.02
Gynecologic	3,113	2,540	(6.4)	573	(5.4)	0.04
Head and neck	2,144	1,711	(4.3)	433	(4.1)	0.01
Hematologic	3,524	2,669	(6.7)	855	(8.1)	0.06
Lung	4,835	3,881	(9.7)	954	(9.0)	0.02
Melanoma	1,426	1,234	(3.1)	192	(1.8)	0.08
Nervous system	2,003	1,701	(4.3)	302	(2.9	0.08
Sarcoma	2,175	1,783	(4.5)	392	(3.7)	0.04
Skin	860	600	(1.5)	260	(2.5)	0.07
Multiple	849	648	(1.6)	201	(1.9)	0.02
Uncertain	303	188	(0.5)	115	(1.1)	0.07
Portal use ≥1 in 365 days before encounter/enrollment	38,546	33,348	(83.4)	5,198	(49.3)	0.77 e

Abbreviations: PROM, patient-reported outcome measures; RUCA, rural–urban commuting area; SDM, standardized mean difference.

a Missing data was <1% for all variables except RUCA (1.4%), race (2.9%), and education (32.8%).

b Standardized differences ≥0.20 and ≥0.50 suggest small and moderate imbalances, respectively.

c Metastatic = ICD-10 codes and natural language processing (NLP) methods agreed cancer was metastatic. Not metastatic = both methods agreed cancer was not metastatic. Indeterminate = the two methods disagreed.

d Method B (single most prevalent type of cancer) is used in this table.

e It denotes SMD ≥ 0.20.

### Secondary Approaches

1. Stage data from the Mayo Clinic *Cancer Registry* were evaluated for the 24,160 patients (47.8% of the cohort) included in this registry.
2. *Treatment plan* was assessed using Beacon (Epic's oncology module), within which clinicians create treatment plans for cancer-directed therapies and order supportive care regimens based on standardized protocols. Within each cancer treatment plan, clinicians must specify a treatment goal. If at least one treatment plan had a goal stated as “Palliative” or “Control,” the cancer was classified as metastatic.
3. Certain *medications* are used in managing specific cancers when that cancer is considered incurable. The linkage of these medications to specific cancers offers another potential method of identifying metastatic disease (see Supplementary Table S1 , available in the online version).
4. Participation in a *phase I clinical trial* may indicate that a patient's cancer is metastatic/incurable. Encounter visits that contained the phrase “Trial” in the encounter description were captured to identify patients seen in the phase I clinical trials clinic.

## Other Variables 8 24

Sociodemographic variables included age, sex, race, ethnicity, education, employment status, marital status, and payor. Rural–urban commuting area (RUCA) codes classify U.S. census tracts using measures of population density, urbanization, and daily commuting. RUCA codes range from 1 to 10 with 1–3, 4–6, and 7–10 representing urban, micropolitan, and rural locations, respectively. Clinical location was coded as tertiary (Mayo Clinic Rochester) or community (all other Mayo Clinic Health System sites). Portal use was defined as the patient having accessed the Epic health care portal at least once in the 12 months prior to first E2C2 contact.

### Statistical Analysis

We summarized the results of all approaches to both cancer site and metastasis determination. The kappa statistic assessed agreement among methods to ascertain cancer site and metastatic disease. Agreement is considered fair for a kappa of 0.21 to 0.40, moderate for a kappa of 0.41 to 0.60, and substantial for a kappa of ≥0.61. 25 In interpreting kappa it is important to note that kappa is not simple agreement but rather the percentage of agreement beyond chance. Thus, a kappa of 0.50 is 50% agreement beyond chance and is therefore considered a moderate level of agreement.

Patient characteristics were described for the full cohort of eligible participants in this stepped wedge trial. Between-group differences were analyzed for symptom report completers versus non-completers (≥1 vs. 0 surveys completed). Imbalance in sociodemographic and clinical characteristics between groups was assessed by examining the *standardized mean difference* (SMD), which is calculated as the difference in means or proportions divided by standard error; imbalance was defined as an absolute value greater than 0.20 26 or 0.25. 27 SMD thresholds were used to assess variable imbalance because SMDs are robust to sample size (unlike statistical significance, which could reflect large absolute differences or just large sample size), and because we had no hypotheses regarding differences between groups.

All analyses were performed in SAS version 9.4 (SAS Institute) and Stata version 18.0 (StataCorp, College Station, Texas, United States). This trial was approved by the Mayo Clinic IRB.

## Results

### Study Sample

Table 2 summarizes the characteristics of the E2C2 eligible cohort ( *n* = 50,559). Most were 40 to 64 years old (42.3%) or ≥65 (51.4%), and 56.4% were women. The sample was predominantly white and non-Hispanic. Most patients had completed education beyond high school, were currently employed or retired, and were married or partnered. About a quarter resided in small town or rural areas (RUCA 7–10). There was a broad distribution of cancer sites with the most common being gastrointestinal (25.3%), breast (21.0%), lung (9.6%), genitourinary (9.0%), hematologic (7.0%), gynecologic (6.2%), sarcoma (4.3%), and head and neck (4.2%). The cancer was determined to be metastatic at some point during the year preceding cohort enrollment or during the 4-year trial interval in 16,544 (32.7%) patients.

Except for a few characteristics, the 40,005 (79.1%) patients who completed at least one symptom report were generally similar to the 10,544 (20.9%) patients who did not complete a report. There were small imbalances (SMD ≥ 0.20) between the two groups for just a few variables, with symptom report completers more likely to have metastatic cancer, higher education, and be receiving antineoplastic therapy. The only moderate imbalance was greater portal use among the symptom report completers (83.4% vs. 49.3%).

### Cancer Site

Table 3 summarizes results of the three operational approaches to categorizing cancer site. Among the total sample, two-thirds (65%; *n* = 33,004) had only 1 cancer diagnosis site identified, approximately one quarter (25%; *n* = 12,673) had 2 sites, and less than 5% (3%; *n* = 4,579) had ≥3 cancer sites identified in the EHR. The site of cancer was nonspecific (not a valid site) in only 303 patients (<0.6%). As shown in Supplementary Table S2 (available in the online version), the approach allowing the two most prevalent ICD-10 cancer site diagnoses per patient (Method C) detected a median of 92% (range, 77% to 98% across cancer sites) of the cases identified by counting all cancer sites (Method A), whereas the approach allowing only the single most prevalent cancer site diagnosis (Method B) identified a median of 65% (range, 13 to 89%). However, the rank order of frequency distributions of specific cancer sites among the three approaches was relatively similar. Moreover, agreement among the three approaches was very good (kappa >0.80) for most cancer sites. Only endocrine and skin cancers had any inter-method kappa <0.7.

**Table 3:** Comparing three approaches for determining type of cancer from EHR

Cancer type	Number of cancer type diagnoses allowed per patient a			Agreement among methods Kappa statistic b			
	Any number	1	1 to 2	A and B	A and C	B and C	A, B, and C
	Method A	Method B	Method C				
	N	N	N				
Breast	11,898	10,611	11,684	0.93	0.99	0.94	0.95
Endocrine	3,474	1,386	3,031	0.55	0.93	0.61	0.72
Gastrointestinal	15,248	12,774	14,735	0.88	0.98	0.90	0.92
Genitourinary	7,007	4,556	6,491	0.76	0.96	0.80	0.85
Gynecologic	3,919	3,113	3,744	0.88	0.98	0.90	0.92
Head and neck	2,954	2,144	2,710	0.83	0.95	0.88	0.89
Hematologic	5,848	3,524	5,348	0.73	0.95	0.78	0.83
Lung	6,669	4,835	6,209	0.82	0.96	0.86	0.88
Melanoma	2,331	1,426	2,010	0.75	0.92	0.82	0.84
Nervous system	3,669	2,003	3,152	0.69	0.92	0.77	0.80
Sarcoma	3,380	2,175	3,060	0.77	0.95	0.82	0.85
Skin, other	6,905	860	5,334	0.20	0.85	0.26	0.50
Multiple c	–	849	–	–	–	–	–
Nonspecific d	303	303	303	–	–	–	–
Total	73,605	50,559	67,811				

a *Method A* counted all categories coded for a patient with no limit on the number of cancer types. *Method B* allowed only one primary diagnosis category per patient. For patients with two or more diagnosis categories, the one that made up >50% of diagnoses was assigned. If there was no majority diagnosis, the category assigned was “multiple.” If there were no specific valid diagnosis categories, the category assigned was “nonspecific.” *Method C* allowed up to two primary diagnosis categories (the two most common out of all that were coded). For those with three or more cancer types, the two most commonly coded cancer type diagnoses were assigned.

b Kappa thresholds of 0.2, 0.4, 0.6, and 0.8 indicate fair, moderate, substantial, and almost perfect agreement.

c When applying Method B, “multiple” was used if there was no majority diagnosis (i.e., one cancer type accounting for >50% of the cancer diagnoses in the EHR).

d Cancer was diagnosed but the cancer type was not specified in the electronic health record.

### Metastatic Disease

Table 4 summarizes the data available for each of the six methods used to classify the patient as having metastatic disease and the pairwise agreement among the methods. Because the phase 1 trial criterion applied to only a small number of patients, we examined in further detail all combinations of the other five methods for determining metastatic disease. Supplementary Table S3 (available in the online version) shows that the number of patients who could be classified as metastatic by one, two, three, four, and all five methods was 10,857, 10,028, 6,824, 3,001, and 590, respectively.

**Table 4:** Comparison of methods to determine metastatic disease

	Primary methods		Secondary methods			
	ICD-10	NLP	Cancer registry	Treatment plan	Medications	Phase I trial a
Metastatic						
• Yes	22,461	22,336	5,914	10,328	5,300	1,050
• No	28,098	28,223	18,246	5,350	43,347	0
• Missing	0	0	26,399	34,881	1,912	49,509
Agreement, kappa b						
• ICD-10 codes	–	–	–	–	–	–
• NLP	0.533	–	–	–	–	–
• Cancer registry	0.217	0.218	–	–	–	–
• Treatment plan	0.400	0.376	0.230	–	–	–
• Medications	0.100	0.103	0.090	0.195	–	–

Abbreviations: EHR, electronic health record; ICD-10, International Statistical Classification of Diseases and Related Health Problems, 10th Revision; NLP, natural language processing.

a Because the completeness and accuracy of EHR data about phase I trial participation was uncertain, agreement with other methods was not calculated.

b Kappa determined in cases for which metastatic status could be classified as “yes” or “no” (excluding “missing”).

ICD-10 codes and NLP data were available for all patients in the trial cohort. Using the ICD-10 approach, 22,461 (44.4%) patients were classified as having metastatic disease compared to 22,336 (44.2%) using the NLP approach. Regarding agreement, 16,554 (32.7%) patients were classified as metastatic by both approaches, 22,333 (44.2%) were classified as non-metastatic by both approaches, and 11,672 (23.1%) were classified as metastatic by one of the approaches but not the other. This resulted in moderate agreement (kappa = 0.53). Notably, this kappa value represents not merely simple agreement but instead 53% agreement over and above chance agreement. Thus, the final method for determining metastasis was agreement between ICD-10 and NLP.

Of the E2C2 cohort, approximately half (47.8%; *n* = 24,160) were in the Mayo Clinic Cancer Registry, and of these 5,914 (24.5%) were classified as having metastatic disease. A Beacon treatment plan with a specific treatment goal was documented for 15,678 (31.0%) patients in the cohort, of which 10,328 (65.9%) had “Palliative” or “Control” selected as the goal. Of the 48,647 patients with medication prescription data, 5,300 (10.9%) had a medication typically used for incurable cancer. Only 1,050 (2.1%) patients had EHR documentation of a phase I trial clinic visit. Following ICD–NLP concordance (kappa = 0.533), the second highest agreement was a Beacon treatment plan which had a kappa of 0.400 with ICD-10, 0.376 with NLP, and 0.230 with the cancer registry.

## Discussion

In our cohort of 50,559 cancer patients participating in a stepped wedge trial of an EHR-facilitated symptom surveillance and management intervention, multiple approaches for determining cancer site and metastasis from EHR data were compared. In addition to the large sample size, strengths of our E2C2 cohort include a diversity of cancer sites, longitudinal measurement of frequently co-occurring symptoms, and the inclusion of participants drawn from both tertiary and community oncology clinics.

Operational rules for how to categorize cancer site are necessary for patients having ICD codes for more than one cancer site. Not surprisingly, the number of cases for specific cancer sites declined when only the one or two most prevalent cancer sites were counted, with the greatest decline being when only the one most prevalent site was counted. However, the relative rank ordering of cancer site frequencies was relatively similar among the three methods. Moreover, inter-method agreement was high (>0.70) for all sites except endocrine and skin cancers; the latter two are less often the most frequently coded cancer when patients have cancer at more than one site. The EHR approach used may depend upon whether one is studying a single versus multiple sites of cancer, the size of the patient sample, and the feasibility of manual chart review for confirmation. Additionally, investigators focusing on subgroups defined by cancer site may prefer to begin with the most sensitive approach: including all qualifying diagnoses, with recognition that adjudication will be required to eliminate false positives.

Determining metastatic status from EHR data is a complex and evolving methodology. The two most common approaches are ICD codes and NLP, used either individually or in combination. 13 14 15 28 29 30 31 Partly this is because, as we observed in our study, these two approaches can be applied in most patients. Also, the ICD–NLP kappa of 0.53 is considered reasonably good inter-method agreement since this is not absolute agreement but the amount of agreement beyond chance (in this case, 53% beyond chance).

We chose a conservative approach by only classifying status as metastatic where both ICD-10 and NLP agreed. Where one approach but not the other indicated metastasis, status was classified as “uncertain” which applied to 23% of our sample. Of interest, ICD-10 and NLP identified a similar number of patients as metastatic and each had a similar level of agreement with the other methods, as shown in Table 4 .

Previous studies evaluating methods for ascertaining metastatic status are detailed in Supplementary Table S4 (available in the online version). Most have focused on a single cancer site, whereas three studies have focused on two to four cancer sites. 28 29 32 Four studies focused principally on detecting cancer recurrence, 28 29 32 33 which can have substantial but not complete overlap with metastasis. All studies used at least two of four methods we studied (ICD, NLP, cancer registries, and/or metastatic-specific drugs), whereas several used claims data. Some studies used medical record review as a gold standard, but this could only be applied to smaller samples, or subsets of subsample of patients. Unlike previous studies, our cohort included a much wider range of cancer sites and compared six different methods.

Cancer registries would seem to be the most reliable and comprehensive source of data, particularly at the time of diagnosis. However, as we observed, many patients with cancer seen at a tertiary center are not necessarily included in that center's registry and, importantly, registry data reflects the stage of cancer at initial diagnosis and is typically not updated to capture the longitudinal trajectory of the disease including progression to more advanced stages. 13 14 15 16 Although Beacon treatment plans can be useful for the subset of patients in which the clinician enters a treatment goal, this Epic module is not used in an important proportion of patients and, when used, a treatment goal may not be listed. Moreover, the absence of a “Palliative” or “Control” treatment goal does not mean that metastatic disease is absent. Finally, this approach is limited to health systems utilizing Epic, though modules with comparable information exist in other EHR systems. Searching prescription data for medications potentially indicative of metastatic disease or attempting to capture phase I trial participation have important limitations for identifying metastatic disease. Many patients with metastatic disease are neither taking these medications nor enrolled in a phase I trial. Other strategies might include claims data, 28 29 information trackers collating data from multiple EHR sources, 34 and machine learning. 31

Using real-world data for research is gaining traction due to large sample sizes, widespread use of EHRs, and the more inclusive nature of the cancer population being investigated. 35 However, problems include incomplete or inaccurate data, varying quality of clinical documentation, challenges of tracking patients longitudinally to assess cancer trajectory, and intricacies of operationalizing clinical variables. 36 The current literature does not outline EHR data best practices for pragmatic trialists, and limitedly describes the methods used in previous studies. 17 18 37 Potential solutions include the design of software for the EHR that enables rapid and standardized reporting of recurrence, common data elements needed for a range of study designs in oncology, use of electronic pathology reports to facilitate collection of recurrence by cancer registries, and mandating by insurers to require reporting of recurrence along with billing codes on medical claims. 16 All require coordination across stakeholders and are potentially resource-intensive.

E2C2 data are from different sources with high heterogeneity. Advanced statistical approaches (e.g., normalization, batch effect removal, imputation) may refine categorization, particularly for variables that are the primary focus of a study rather than covariates. Also, large language model advances are promising 21 38 39 and may be integrated into future specification of EHR-derived cancer characteristics, although some are limited to specific cancer sites or data sources. Challenges of using real-world EHR data have been articulated and call out for examining new strategies. 17 36

Deciding what is “good enough” covariate adjudication, i.e., fitness for use, 18 is a highly pragmatic consideration as research migrates along the translational continuum to population-level deployment. For example, the approaches described in this paper may be sufficient when describing characteristics of a sample or when adjusting for covariates. However, a pragmatic trial evaluating treatments to decrease progression to metastasis within a single type of cancer may warrant more rigorous methods including detailed chart review to ascertain the presence of metastasis as a primary outcome.

An important caveat is the predominantly white, non-Hispanic sample, which may limit generalizability of our results to racially and socioeconomically diverse populations. At the same time, our cohort's demographic characteristics are representative of the upper Midwest general population. A salient advantage is that we are able to generalize to rural patients (who face care access barriers) given that a quarter of the cohort resided in rural areas.

## Conclusion

Strengths of this pragmatic trial include the large sample size, broad range of cancer sites and treatments, inclusion of a substantial portion of patients with metastatic disease and notable symptom burden, and the use of robust strategies for clinical characterization. Our findings can inform future use of EHR data for classifying cancer site and metastatic status in research, including pragmatic trials of novel healthcare delivery interventions as well as clinical epidemiological studies.

## Clinical Relevance Statement

The combination of ICD diagnostic codes and natural language processing is a more sensitive approach to detecting the potential presence of metastasis than cancer register, treatment plans, or medication data.

## Multiple-Choice Questions

1. Using electronic health records (EHR), which two methods in combination allow classification of metastatic status for the greatest proportion of patients with cancer and with the highest agreement.
  - ICD diagnostic codes and cancer registry data
  - Cancer registry data and natural language processing
  - ICD diagnostic codes and natural language processing
  - Natural language processing and Beacon treatment plans
  **Correct Answer:** The correct answer is option c. Nearly all patients have ICD diagnostic codes in the EHR, and natural language processing is likewise applicable to all patients.
2. For patients with more than one cancer site coded in the EHR, counting only the most frequently coded cancer would have the lowest sensitivity for patients with:
  - Lung cancer
  - Skin cancer, non-melanoma
  - Sarcoma
  - Melanoma
  **Correct Answer:** The correct answer is option b. Compared to counting all cancer diagnoses in the EHR, counting only the most frequently coded cancer would miss 80 to 90% of non-melanoma skin cancers. Another cancer site with a high non-detection rate would be endocrine cancer.
