# Supplementary material for: Using Electronic Health Records to Classify Cancer Site and Metastasis
Source: Appl Clin Inform. 2025 Jun 18;16(3):556–68. doi: 10.1055/a-2544-3117 (PMC12176508; doi:10.1055/a-2544-3117)
Supplement: Supplementary file 1 — Supplementary Material [file 10-1055-a-2544-3117-s202412ra0404.pdf]

Supplementary Table S1 Medications potentially indicative of incurable cancer[a]

Cancer type	Medications
Breast	Everolimus, lapatinib ditosylate, palbociclib, ribociclib succinate, ribociclib succinate/letrozole
Head and neck	Abiraterone acetate, submicronized, abiraterone acetate, afatinib dimaleate, bicalutamide, enzalutamide, lenvatinib mesylate
Genitourinary	Axitinib, belzutifan, cabozantinib s-malate, erdafitinib, everolimus, lenvatinib mesylate, olaparib, pazopanib HCl, pemigatinib, rucaparib camsylate, sorafenib tosylate, tivozanib HCl
Prostate	Abiraterone acetate, submicronized, abiraterone acetate, apalutamide, darolutamide, enzalutamide
Sarcoma	Avapritinib, palbociclib, pazopanib HCl, regorafenib, ripretinib, sunitinib malate, tazemetostat hydrobromide, temozolomide
Lung	Adagrasib, ado-trastuzumab emtansine, afatinib dimaleate, alectinib HCl, amivantamab-vmjw, bevacizumab, bevacizumab-adcd, bevacizumab-awwb, bevacizumab-bvzr, brigatinib, cabozantinib s-malate, capmatinib hydrochloride, cemiplimab-rwlc, ceritinib, cetuximab, crizotinib, dabrafenib mesylate, doxorubicin HCl, doxorubicin HCl peg-liposomal, durvalumab, entrectinib, erlotinib HCl, everolimus, fam-trastuzumab deruxtecn-nxki, gefitinib, ipilimumab, lanreotide acetate, larotrectinib sulfate, lorlatinib, lurbinectedin, mobocertinib succinate, octreotide acetate, octreotide acetate, microspheres, pralsetinib, ramucirumab, selpercatinib, sotorasib, temozolomide, tepotinib HCl, topotecan HCl, trametinib dimethyl sulfoxide, vinorelbine tartrate
Lymphoma	Acalabrutinib, acalabrutinib maleate, duvelisib, everolimus, ibrutinib, lenalidomide, nivolumab-relatlimab-rmbw, selinexor, tazemetostat hydrobromide, venetoclax, zanubrutinib
Gastrointestinal	Binimetinib, cabozantinib s-malate, encorafenib, lapatinib ditosylate, lenvatinib mesylate, olaparib, regorafenib, sorafenib tosylate, trifluridine/tipiracil HCl, tucatinib
Primary Brain[b]	Lomustine, procarbazine HCl, temozolomide
Leukemia[b]	Azacitidine, decitabine, decitabine/cedazuridine, venetoclax

Notes: [a]Based upon expert consensus and NCCN guidelines.
[b]These drugs for primary brain tumors or leukemia may also be used in curative-intent settings.

Supplementary Table S2 Differences in number of cases identified by the three EHR methods for determining cancer site

	Number of cancer type diagnoses allowed per patient					Identification rate compared to method A[a]		
Cancer type	Any number		1		1 to 2	1	1 to 2	
	Method A		Method B		Method C	Method B	Method C	
	N (Rank)		*N* (Rank)		*N* (Rank)	%	%	
Breast	11,898	(2)	10,611	(2)	11,684	89.2	98.2	(2)
Endocrine	3,474	(9)	1,386	(11)	3,031	39.9	87.2	(10)
Gastrointestinal	15,248	(1)	12,774	(1)	14,735	83.8	94.3	(1)
Genitourinary	7,007	(3)	4,556	(4)	6,491	65.0	92.6	(3)
Gynecologic	3,919	(7)	3,113	(6)	3,744	79.5	95.5	(7)
Head and neck	2,954	(11)	2,144	(8)	2,710	72.6	91.7	(11)
Hematologic	5,848	(6)	3,524	(5)	5,348	60.3	91.5	(5)
Lung	6,669	(5)	4,835	(3)	6,209	72.6	93.1	(4)
Melanoma	2,331	(12)	1,426	(10)	2,010	61.2	86.2	(12)
Nervous system	3,669	(8)	2,003	(9)	3,152	54.5	85.9	(8)
Sarcoma	3,380	(10)	2,175	(7)	3,060	64.3	90.5	(9)
Skin, other	6,905	(4)	860	(12)	5,334	12.5	77.2	(6)
Median % (range)						64.7 (12.5–89.2)	91.6 (77.2–98.2)	

 © 2025. The Author(s).

Supplementary Table S3 Metastatic disease by all possible combinations of five methods

	Method					
**Method**	**1**	**2**	**3**	**4**	**5**	**Number**
**Combinations**	**ICD-10**	**NLP**	**Cancer registry**	**Treatment plan**	**Medications**	**positive**
Metastatic "yes" by each method, N[a]	22,461	22,336	5,914	10,328	5,300	
5 methods positive						**590**
12345	+	+	+	+	+	590
4 methods positive						**3,001**
1234	+	+	+	+	–	1,360
1235	+	+	+	–	+	309
1245	+	+	–	+	+	1,265
1345	+	–	+	+	+	21
2345	–	+	+	+	+	46
3 methods positive						**6,824**
123	+	+	+	–	–	1,533
124	+	+	–	+	–	3,642
125	+	+	–	–	+	970
134	+	–	+	+	–	116
135	+	–	+	–	+	26
145	+	–	–	+	+	108
234	–	+	+	+	–	154
235	–	+	+	–	+	40
245	–	+	–	+	+	177
345	–	–	+	+	+	58
2 methods positive						**10,028**
12	+	+	–	–	–	6,901
13	+	–	+	–	–	245
14	+	–	–	+	–	496
15	+	–	–	–	+	252
23	–	+	+	–	–	486
24	–	+	–	+	–	500
25	–	+	–	–	+	183
34	–	–	+	+	–	250
35	–	–	+	–	+	31
45	–	–	–	+	+	684
1 method positive						**10,857**
1	+	–	–	–	–	4,627
2	–	+	–	–	–	4,180
3	–	–	+	–	–	649
4	–	–	–	+	–	861
5	–	–	–	–	+	540
0 methods positive	–	–	–	–	–	**19,259**
≥1 method positive						31,300

Abbreviations: ICD-10, International Statistical Classification of Diseases and Related Health Problems, 10th Revision; NLP, natural language processing.
Note:Bold values are statistically significant.
[a]Total *N* for each method equals the number of patients in whom the method indicated metastatic disease. In this table, that is the sum of *N*'s for the rows where that method had a + sign. For example, the ICD-10 method indicated metastatic disease in 22,461 patients (which is the sum of 590 where all five methods were positive, 2,955 where four methods are positive, 6,395 where three methods are positive, 7,894 where two methods are positive, and 4,627 where one method is positive.

 © 2025. The Author(s).

Supplementary Table S4 Synopsis of previous studies using electronic data sources for identifying metastatic cancer

Author (Year)	Ref	*N*	Cancer	Data	ICD	NLP	Reg	Claim	Rx	Comments
Alba et al (2021)	14	76,082	Prostate	VA CDW	X	X	X		X	NLP identified largest number of cases; VA registry too few cases (did not update data longitudinally); drugs not specific but also used to treat aggressive local cancers
Carroll et al (2019)	29	659 colon<br>280 lung<br>2,053 breast	Colon<br>Lung<br>Breast	1-Can CORS<br>2-CRN	X		X	X	X	*Recurrence* detection; similar approach to Hassett et al (2017)[28] and Ritzwoller et al (2018)[33] applied to new cohort; updated for ICD-10; sensitivity and specificity of ICD codes varied widely by cancer site
Choi et al (2021)	40	7,917 claims<br>7,087 EHR	Lung	Op-tum	X	X		X	X	Non-small-cell lung cancer—required ICD codes for metastasis + specific drugs (both required) + NLP
Hassett et al (2014)	32	5,298 CRN<br>929 Can-CORS	Colon<br>Lung<br>Breast<br>Prostate	1-Can CORS<br>2-CRN	X		X	X		*Recurrence* detection; linked Medicare claims and/or EHR data to two types of cancer registries; medical record review was gold standard for recurrence; sensitivity and specificity of ICD codes varied widely by cancer site
Hassett et al (2017)	28	1,100 lung<br>3,427 colon	Lung<br>Colon	1-Can CORS<br>2-CRN	X		X	X	X	*Recurrence* detection; linked Medicare claims and/or EHR data to two types of cancer registries; algorithms using ICD (for secondary malignancy) and treatment (chemo or radiation) codes; used statistical modeling
He et al (2019)	30	1,727	Melanoma	INPC	X	X	X		X	Structured EHR algorithm = ICD code for melanoma + ICD or local code for metastasis or melanoma<br>NLP superior to structured algorithm (2x as many cases) using chart review gold standard; only 246 in registry
Ling et al (2019)	13	1,886	Breast	Stanford	X	X	X			Most (73%) were not metastatic at baseline (registry); median f/u = 9 years; chart review in sample of 146<br>Coupled ICD/drug/procedure codes with NLP
Ritzwoller et al (2018)	33	10,701	Breast	CRN	X		X	X	X	*Recurrence* detection; similar approach to Hassett et al (2017)[28] but applied to breast cancer
Seneviratne (2018)	31	327 of 5,861	Prostate	Stanford	X	X	X			Machine learning with all structured variables better than ICD or NLP with registry initial staging as gold standard
Stafkey-Mailey et al (2013)	41	740	Lung		X	X				Abstract. 60% identified by ICD and 40% more by NLP; chart review gold standard confirmed 87% as metastatic
Ping et al (2013)	34	152	Liver	Taiwan						Information tracker from multiple reports ($\mu = 30$/pt over $\mu = 5$ yr): radiology (53%), ultrasound (24%), discharge (14%), pathology (5%), admission (2%), operation (2%)
Warner et al (2016)	15	2,323	Lung	Van-derbilt		X	X			Concordance of NLP and registry in stage IIIA/B = 75%; 1,025 of 2,323 patients (44%) were in cancer registry

Abbreviations: CanCORS, Cancer Care Outcomes Research and Surveillance (CanCORS) Consortium large cohort study; CDW, Corporate Data Warehouse; CRN, Cancer Research Network, a consortium of health maintenance organizations (HMO) affiliated with the HMO Research Network (HMORN) and the NCI; Reg, registry; Rx, metastatic-specific drugs; VA, Veteran Affairs Corporate Data Warehouse.

 © 2025. The Author(s).
